# Prenatal ethanol exposure alters adult hippocampal VGLUT2 expression with concomitant changes in promoter DNA methylation, H3K4 trimethylation and miR-467b-5p levels

**DOI:** 10.1186/s13072-015-0032-6

**Published:** 2015-09-28

**Authors:** Christine R. Zhang, Mei-Fong Ho, Michelle C. Sanchez Vega, Thomas H. J. Burne, Suyinn Chong

**Affiliations:** Mater Research Institute, The University of Queensland, Translational Research Institute, Level 4, 37 Kent St, Woolloongabba, QLD 4102 Australia; Queensland Brain Institute, The University of Queensland, St Lucia, QLD 4072 Australia

**Keywords:** Developmental programming, DNA methylation, Histone modifications, Ethanol exposure, MicroRNA

## Abstract

**Background:**

Maternal consumption of alcohol during pregnancy is associated with a range of physical, cognitive and behavioural outcomes in the offspring which are collectively called foetal alcohol spectrum disorders. We and others have proposed that epigenetic modifications, such as DNA methylation and post-translational histone modifications, mediate the effects of prenatal alcohol exposure on gene expression and, ultimately, phenotype. Here we use an inbred C57BL/6J mouse model of early gestational ethanol exposure equivalent, developmentally, to the first 3–4 weeks of pregnancy in humans to examine the long-term effects on gene expression and epigenetic state in the hippocampus.

**Results:**

Gene expression analysis in the hippocampus revealed sex- and age-specific up-regulation of *solute carrier family 17 member 6* (*Slc17a6*), which encodes vesicular glutamate transporter 2 (VGLUT2). Transcriptional up-regulation correlated with decreased DNA methylation and enrichment of histone H3 lysine 4 trimethylation, an active chromatin mark, at the *Slc17a6* promoter. In contrast to *Slc17a6* mRNA levels, hippocampal VGLUT2 protein levels were significantly decreased in adult ethanol-exposed offspring, suggesting an additional level of post-transcriptional control. MicroRNA expression profiling in the hippocampus identified four ethanol-sensitive microRNAs, of which miR-467b-5p was predicted to target *Slc17a6.* In vitro reporter assays showed that miR-467b-5p specifically interacted with the 3′UTR of *Slc17a6*, suggesting that it contributes to the reduction of hippocampal VGLUT2 in vivo. A significant correlation between microRNA expression in the hippocampus and serum of ethanol-exposed offspring was also observed.

**Conclusions:**

Prenatal ethanol exposure has complex transcriptional and post-transcriptional effects on *Slc17a6* (VGLUT2) expression in the mouse hippocampus. These effects are observed following a relatively moderate exposure that is restricted to early pregnancy, modelling human consumption of alcohol before pregnancy is confirmed, and are only apparent in male offspring in adulthood. Our findings are consistent with the idea that altered epigenetic and/or microRNA-mediated regulation of glutamate neurotransmission in the hippocampus contributes to the cognitive and behavioural phenotypes observed in foetal alcohol spectrum disorders. Although further work is needed in both mice and humans, the results also suggest that circulating microRNAs could be used as biomarkers of early gestational ethanol exposure and hippocampal dysfunction.

**Electronic supplementary material:**

The online version of this article (doi:10.1186/s13072-015-0032-6) contains supplementary material, which is available to authorized users.

## Background

Structural and functional abnormalities of the central nervous system are commonly observed in foetal alcohol spectrum disorders [1]. Cognitive and behavioural outcomes in individuals exposed to alcohol in utero include intellectual impairment, adaptive dysfunction and deficits in executive functioning, learning and memory [2]. The hippocampus has received considerable attention in studies of gestational alcohol exposure due to its sensitivity to alcohol and its role in learning and memory. Alcohol-associated reductions in hippocampal volume have been reported in humans exposed in utero, and combined imaging and behavioural studies have linked altered hippocampal volume with memory deficits [3, 4]. Animal studies have similarly documented ethanol-induced reductions in hippocampal volume [5, 6] and impaired hippocampal-dependent learning and memory [7, 8]. Further, they have revealed that prenatal ethanol exposure results in altered electrophysiological properties [9, 10], as well as cell loss [6] and compromised neurogenesis [11, 12] in the hippocampus. Dysregulation of a number of genes involved in glutamatergic signalling and synaptic plasticity, including *vesicular glutamate transporter 1*, *complexin 1*, *excitatory amino acid transporters 1* and *3*, and some *N*-methyl-d-aspartate (NMDA) receptor subunits, has also been reported in rats [7, 13, 14] but the mechanisms underlying these ethanol-induced expression changes in the hippocampus are not known.

Epigenetic modifications, including DNA methylation and post-translational histone modifications which package the DNA into chromatin, play an important role in development by regulating transcription. MicroRNAs (miRNAs), small ~22 nucleotide non-coding RNAs, also fine-tune gene expression either by destabilising target mRNAs or blocking translation [15]. DNA methylation, post-translational histone modifications and miRNAs have received increasing attention as potential mediators of the effects of environmental exposures on phenotype and disease risk. Consistent with this, global levels of DNA methylation as well as histone H3 lysine 9 dimethylation and histone H3 lysine 27 dimethylation were found to be disrupted in the hippocampus following neonatal ethanol exposure in rats [16] and C57BL/6J mice [17], respectively. Recent studies have further linked ethanol-induced changes in histone H4 lysine 8 acetylation [18] and DNA methylation [19] with altered hippocampal gene expression in C57BL/6J mice. There is evidence that ethanol exposure can alter miRNA expression in cortical neuroepithelial precursor cells in culture [20] and C57BL/6J whole brain [21–23], but its impact on the hippocampus has not been fully explored [24].

To date, most studies of the effects of prenatal ethanol exposure on the hippocampus have used exposures which either extended throughout pregnancy or were equivalent to the third trimester of pregnancy in humans. Evidence that the majority of women cease alcohol consumption when pregnancy is confirmed (~5 weeks gestation) [25] highlights a need for investigations that target early gestational ethanol exposure. This window of exposure incorporates early neurulation and, crucially, embryonic epigenetic reprogramming, when dynamic changes in epigenetic state are occurring genome-wide. We previously established an inbred C57BL/6J mouse model of prenatal ethanol exposure that is based on voluntary maternal consumption of 10 % ethanol (v/v) from 0.5 to 8.5 days post coitum (dpc) [26]. The estimated peak maternal blood alcohol concentration is relatively moderate at 120 mg/dl or 0.12 % [26] and the exposure period is equivalent, developmentally, to the first 3–4 weeks of a human pregnancy. We found that ethanol-exposed offspring displayed significant alterations in behaviour [27], craniofacial structure and an increased probability of transcriptional silencing at the epigenetically sensitive *Agouti viable yellow* (*A*^*vy*^) allele [26]. Here we examine *solute carrier family 17 member 6* (*Slc17a6*), which plays a role in excitatory neurotransmission, synaptic plasticity and cognition [28], and describe ethanol-induced changes in hippocampal expression that are linked to altered promoter DNA methylation, post-translational histone modifications and miRNA levels.

## Results

### Prenatal ethanol exposure disrupts the developmental silencing of *Slc17a6* in the male hippocampus

The average daily consumption of 10 % (v/v) ethanol by pregnant C57BL/6J females was 3.3 ± 0.7 ml, which was not significantly different to the volume of water consumed by control mice (Additional file 1: Figure S1A). There was no effect of ethanol on maternal body weight gain during the exposure period (Additional file 1: Figure S1B) or litter size at weaning (Additional file 1: Figure S1C), indicating that the exposure had no significant impact on maternal health or offspring viability.

**Fig. 1:**
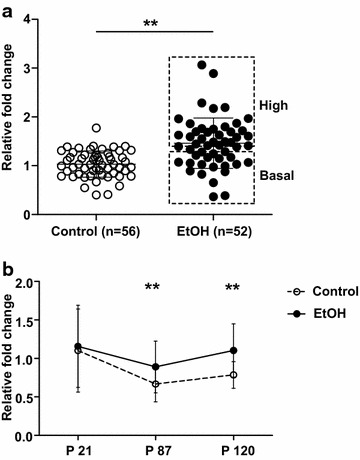
qPCR analysis of *Slc17a6* expression in the male hippocampus. **a** *Slc17a6* expression in the male hippocampus at P87. A total of 56 control mice and 52 ethanol-exposed (EtOH) mice from three independent cohorts were assayed. Basal (fold change of ~1 relative to controls) and high (>1.35-fold) *Slc17a6* expressors in the ethanol-exposed group are *boxed*. **b** Hippocampal *Slc17a6* expression in males at different ages. Controls (*open circle*) at P21 (*n* = 30 mice), P87 (*n* = 56 mice) and P120 (*n* = 11 mice) and ethanol-exposed mice (*filled circle*) at P21 (*n* = 36 mice), P87 (*n* = 52 mice), P120 (*n* = 18 mice) are shown. Data were normalised to *Hprt1* and are plotted as mean and standard deviation. **P < 0.01 (*t* test, two-tailed)

To examine the long-term effects of prenatal ethanol exposure on the hippocampal transcriptome, microarray-based gene expression analyses were performed on adult male hippocampi [postnatal day (*P*) 87, *n* = 6 per group]. Only four genes, *indolethylamine N*-*methyltransferase* (*Inmt*), *melanoma inhibitory activity 1* (*Mia1*), *solute carrier family 17 member 6* (*Slc17a6*) and *teashirt zinc finger family member 2* (*Tshz2*), were found to be differentially expressed (>1.5-fold, uncorrected *P* ≤ 0.05) in ethanol-exposed mice compared to controls (Additional file 1: Table S1). None of these genes passed multiple testing correction suggesting that they may be false positives; however, qPCR analysis of hippocampal *Slc17a6* expression in additional cohorts of mice confirmed the significant overall up-regulation of this gene in the ethanol-exposed group (Fig. 1a). Interestingly, not all ethanol-exposed offspring were equally affected; the highest *Slc17a6* expressors were increased approximately threefold compared to controls while others had basal levels of expression which were equivalent to the controls. This variation in expression (*F* test, *P* < 0.001), even within litters, in inbred ethanol-exposed mice parallels our previous findings with *A*^*vy*^ [25] lending further support to the idea that the influence of early gestational ethanol exposure on gene expression is stochastic. Altered expression of *Slc17a6* was not detected in adult ethanol-exposed female offspring, indicating that the effect is also sex-specific (Additional file 1: Figure S2).

*Slc17a6* (VGLUT2) is one of three vesicular glutamate transporters that function in the uptake of glutamate into synaptic vesicles. It is primarily expressed in the hippocampus during the first 2–3 weeks of postnatal development, coincident with brain growth and maturation [29, 30]. A developmental switch in expression then occurs in which VGLUT2 is downregulated and VGLUT1 is up-regulated to become the predominant VGLUT in the hippocampus in adulthood [29, 30]. Consistent with the literature, qPCR analysis in control male offspring showed a decrease in hippocampal *Slc17a6* expression from P21 (3 weeks of age) to P87 (adulthood), which persisted until at least P120 (Fig. 1b). Expression of *Slc17a6* in ethanol-exposed male offspring was similar to controls at P21, but was significantly increased at P87 and P120 (Fig. 1b; Additional file 1: Figure S3), indicating that prenatal ethanol exposure disrupts the developmental silencing of *Slc17a6* in the hippocampus. Expression of *Slc17a7* (VGLUT1) was not changed in the hippocampi of adult ethanol-exposed offspring in the initial microarray experiment, so was not examined further.

### Increased transcription of *Slc17a6* correlates with decreased promoter DNA methylation in adult ethanol-exposed offspring

**Fig. 2:**
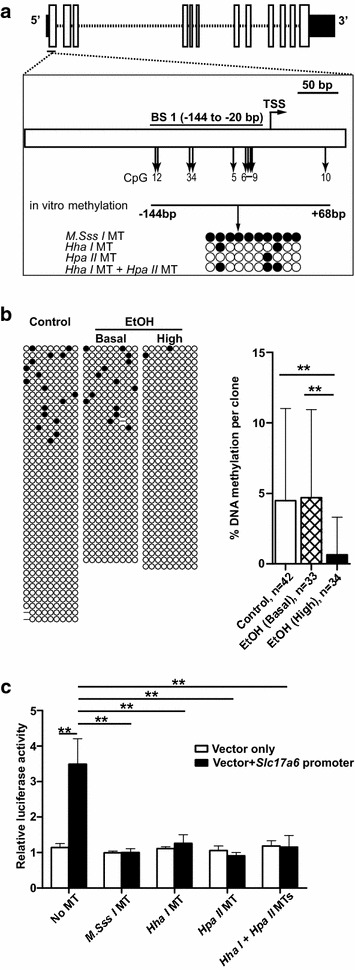
DNA methylation at the *Slc17a6* promoter. **a** Schematic representation of the *Slc17a6* gene. *Solid boxes* represent the 5′ and 3′UTRs and *open boxes* represent exons. A region spanning −255 to +90 bp relative to the transcriptional start site (TSS) is drawn below. The region (BS1, −144 to −20 bp) and CpG sites (*vertical arrows*, CpGs 1–9) examined by clonal bisulphite sequencing are indicated, as is the region used in in vitro reporter assays (CpGs 1–10, −144 bp to +68 bp). Four different CpG methylation patterns were generated for reporter assays using three bacterial methyltransferases (MTs), *M.SssI* MT, *Hha*I MT and *Hpa*II MT either alone or in combination. *Filled* and *open circles represent* methylated and unmethylated CpGs, respectively. **b** DNA methylation in the adult hippocampus (P87 and 120). Each line of nine CpGs represents the DNA methylation state of one allele in one cell. Clones from controls (total *n* = 42 from 16 mice) as well as basal (total *n* = 33 from 16 mice) and high (total *n* = 34 from 17 mice) *Slc17a6* expressors in the ethanol-exposed (EtOH) group are shown. A *graph* of % DNA methylation per clone is also shown. **c** The impact of *Slc17a6* promoter methylation (CpGs 1–10, −144 to +68 bp) on luciferase activity in vitro. The results of three independent experiments in CAD cells are shown. Data are presented as mean and standard deviation. **P < 0.01 (*t* test, two-tailed)

To examine whether *Slc17a6* expression was associated with a specific promoter DNA methylation pattern, clonal bisulphite sequencing was carried out on a region extending from −144 to −20 bp relative to the transcriptional start site identified by Li and colleagues in the mouse brain [31]. The region (BS1) contains nine CpG dinucleotides (Fig. 2a). Adult hippocampal samples were divided into three groups for DNA methylation analysis: controls, ethanol-exposed offspring that had basal expression of *Slc17a6* (fold change ~1 relative to controls), and ethanol-exposed offspring that had increased expression of *Slc17a6* (>1.35-fold compared to controls). The rationale for separating ethanol-exposed mice into two subgroups, representing unaffected offspring (basal expressors) and affected offspring (high expressors), was to reduce background “noise” and allow epigenetic changes that are associated with transcriptional up-regulation to be more easily detected. Ethanol-exposed basal *Slc17a6* expressors had an average of 4.7 % methylation per clone which was not significantly different from controls (average of 4.5 % methylation per clone) (Fig. 2b). In contrast, ethanol-exposed high *Slc17a6* expressors displayed a significant decrease in promoter DNA methylation, with an average of 0.7 % methylation per clone (Fig. 2b). At P21, where hippocampal *Slc17a6* transcription is high and there is no differential expression between groups, the average % methylation per clone was not significantly different between control and ethanol-exposed offspring (Additional file 1: Figure S4).

An in vitro reporter assay was used to investigate whether DNA methylation at the *Slc17a6* promoter was capable of affecting gene expression. The promoter region, spanning −144 to +68 bp and containing ten CpG dinucleotides (nine of which were examined previously by clonal bisulphite sequencing) was inserted upstream of a firefly luciferase reporter gene. Three bacterial methyltransferases, including a CpG methyltransferase (*M.Sss I*), *Hpa*II methyltransferase and *Hha*I methyltransferase, were then used either alone or in combination to generate four different promoter methylation patterns prior to transient transfection into a murine neuronal cell line, Cath.a-differentiated (CAD). The CAD cell line is derived from a CNS catecholaminergic cell line, Cath.a, and expresses markers found in differentiated neurons [32]. Transfection efficiency was normalised by co-transfection with a plasmid containing *Renilla* luciferase. Figure 2c shows that insertion of the *Slc17a6* promoter region upstream of the reporter gene significantly increased luciferase activity compared to vector only (*P* < 0.01), confirming its promoter activity. DNA methylation had no effect on the luciferase activity of vector-only constructs, but it significantly reduced luciferase activity in constructs containing the *Slc17a6* promoter (*P* < 0.01, Fig. 2c). Moreover, the effect of DNA methylation was the same regardless of whether all ten CpGs in the *Slc17a6* promoter were methylated (*M.Sss I*) or only one CpG site was methylated (*Hpa*II methyltransferase). Similar results were obtained with a second neuronal cell line, Neuro 2a (Additional file 1: Figure S5). The results indicate that small changes in DNA methylation can have a dramatic effect on *Slc17a6* promoter function.

### Enrichment of histone H3 lysine 4 trimethylation (H3K4me3), an active chromatin mark, at *Slc17a6* is associated with increased transcription

**Fig. 3:**
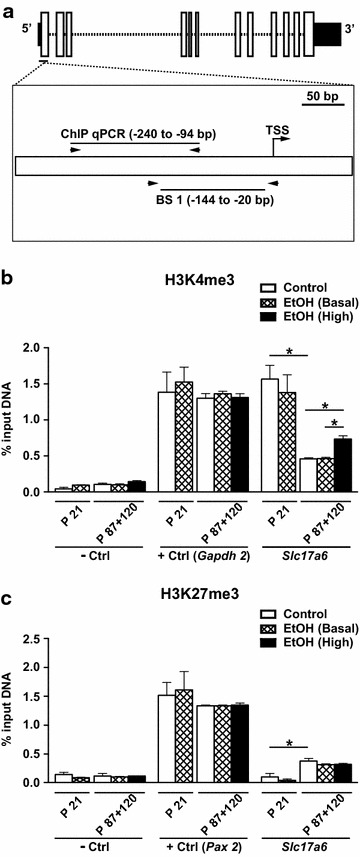
Analysis of H3K4me3 and H3K27me3 at *Slc17a6.* **a** Schematic representation of the *Slc17a6* gene showing the region assayed by ChIP-qPCR (−240 to −94 bp) relative to BS1 and the TSS is shown. *Horizontal arrows* represent PCR primers. **b** H3K4me3 in the male hippocampus at P21 and P87 + 120 (*n* = 12 mice/group, pooled). A gene desert and *Gapdh2* were used as a negative control (−Ctrl) and positive control (+Ctrl), respectively. **c** H3K27me3 in the male hippocampus at P21 and P87 + 120 (*n* = 12 mice/group, pooled). A gene desert and *Pax2* were used as negative and positive controls, respectively. Data are shown as mean and standard deviation. *P < 0.05 (*t* test, two-tailed)

ChIP-qPCR was used to assay the hippocampal levels of H3K4me3 (associated with transcriptional activation) and histone H3 lysine 27 trimethylation (H3K27me3, associated with transcriptional repression) at a region in the *Slc17a6* promoter (−240 to −94 bp; Fig. 3a) that has previously been shown to be enriched for these marks in adult C57BL/6 cerebellum [33, 34]. Chromatin was prepared from the pooled hippocampi of twelve males per group. H3K4me3, but not H3K27me3, was enriched at *Slc17a6* in both ethanol-exposed and control offspring at P21, consistent with the high expression of *Slc17a6* in both groups at this age (Fig. 3b, c). In adult control offspring, H3K4me3 was significantly decreased and H3K27me3 was significantly increased compared to P21 (Fig. 3b, c) coincident with the developmental silencing of *Slc17a6*. H3K4me3 and H3K27me3 levels in adult ethanol-exposed basal *Slc17a6* expressors were no different to controls; however, ethanol-exposed high *Slc17a6* expressors had a significant increase in H3K4me3 compared to controls (Fig. 3b, c).

### Prenatal ethanol exposure is associated with a reduction in hippocampal VGLUT2 levels

**Fig. 4:**
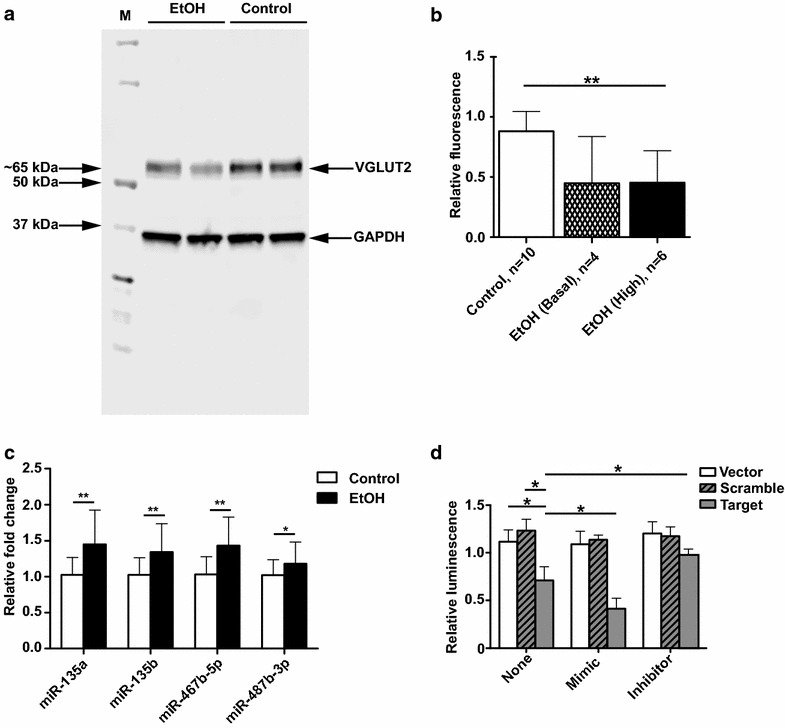
Post-transcriptional regulation of *Slc17a6.* **a** Representative western blot of VGLUT2 (65 kDa) in the adult male hippocampus (*n* = 2 per group). GAPDH (38 kDa) was used as a loading control. **b** Quantitation of VGLUT2 levels in controls (*n* = 10 mice), basal expressors of *Slc17a6* in the ethanol-exposed group (*n* = 4 mice) and high expressors of *Slc17a6* in the ethanol-exposed group (*n* = 6 mice). **c** qPCR analysis of miRNA expression in the adult male hippocampus (*n* = 28 in the ethanol-exposed group and *n* = 32 in the controls). Expression was normalised to U6 snRNA. **d** miR-467b-5p targets the 3′UTR of *Slc17a6* in an in vitro reporter assay. Luminescence is shown for vector only, vector containing the 3′UTR of *Slc17a6* (Target) and vector with a mutated *Slc17a6* 3′UTR (Scramble) under three different co-transfection conditions: no co-transfection (None), addition of a miR-467b-5p mimic (Mimic) or addition of a miR-467b-5p inhibitor (Inhibitor). Assays were done in quadruplicate, and the results from three independent experiments in CAD cells are shown. Data are shown as mean and standard deviation. *P < 0.05, **P < 0.01 (*t* test, two-tailed)

Western blotting was used to investigate whether *Slc17a6* mRNA levels correlated with VGLUT2 protein levels in the adult male hippocampus (Fig. 4a). We found that, compared to controls, VGLUT2 was significantly lower in high *Slc17a6* expressors in the ethanol-exposed group, and showed a trend towards reduced levels (*P* = 0.06) in basal *Slc17a6* expressors in the ethanol-exposed group (Fig. 4b). Pearson correlation analysis found no relationship between *Slc17a6* mRNA and VGLUT2 protein levels in the ethanol-exposed group (*P* > 0.05). The results indicate that prenatal ethanol exposure is associated with a reduction in VGLUT2 protein regardless of the *Slc17a6* mRNA levels in hippocampus. Moreover, they suggest that there is post-transcriptional regulation of VGLUT2.

### Alterations in adult hippocampal miRNA expression in response to prenatal ethanol exposure

Expression profiling was performed in the adult hippocampus (*n* = 6 per group) and of 944 mature miRNAs assayed, 488 were expressed in both the ethanol-exposed and control groups. Of these, fifteen miRNAs were differentially expressed (>1.5-fold, uncorrected *P* < 0.0005, Additional file 1: Table S2). Seven miRNAs were selected for qPCR validation experiments based on factors such as expression fold change, *P* value, miRNA family membership and genomic location (Additional file 1: Table S2). Four out of seven miRNAs, miR-135a, miR-135b, miR-467b-5p and miR-487b, were confirmed to be significantly up-regulated in ethanol-exposed mice (Fig. 4c).

### Ethanol-sensitive miR-467b-5p targets *Slc17a6*

One of the ethanol-sensitive miRNAs, the mouse-specific miR-467b-5p, was predicted to bind to the 3′UTR of *Slc17a6* by TargetScan (Release 6.2) [35]. Therefore, the interaction was tested experimentally using an in vitro reporter assay. Briefly, the predicted miR-467b-5p target site (position 374-380 of *Slc17a6* 3′UTR, NCBI:NM_080853) or a mutated target site (Scramble) was inserted into the 3′UTR of a firefly luciferase reporter gene. The constructs were then transiently transfected into a murine neural cell line, CAD, which is known to express miR-467b-5p (determined by qPCR). The specificity of the interaction was tested by co-transfection with either a miR-467b-5p mimic or inhibitor. Figure 4d shows that firefly luciferase activity was significantly decreased when the putative target site of miR-467b-5p was inserted adjacent to the reporter compared to vector only or the Scramble construct. Furthermore, the decrease in luminescence was augmented by co-transfection of a miR-467b-5p mimic and partially ameliorated by co-transfection of a miR-467b-5p inhibitor (Fig. 4d). There was no effect of the mimic or inhibitor on the vector only or Scramble constructs. Similar results were obtained with a second neuronal cell line, Neuro 2a (Additional file 1: Figure S6). Taken together, the results indicate a specific interaction between miR-467b-5p and the 3′UTR of *Slc17a6* in vitro, and suggest that a similar interaction contributes to reduced hippocampal VGLUT2 protein levels in ethanol-exposed adult males in vivo.

### Ethanol-sensitive miRNA expression in the hippocampus is mirrored in the serum of the same animal

**Fig. 5:**
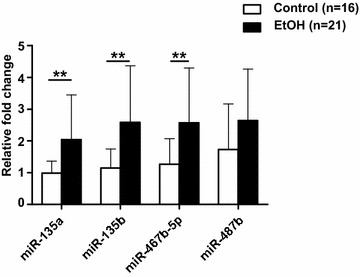
Ethanol-sensitive miRNA expression in the serum of adult male offspring. qPCR analysis of ethanol-sensitive miRNAs in the serum of adult (PD 87 and 120) male ethanol-exposed (*n* = 21 mice) and control (*n* = 16 mice) offspring from three independent cohorts. Expression was normalised to exogenously spiked synthetic *C. elegans* miR-39 (Syn-cel-miR-39). Data are shown as mean and standard deviation. **P < 0.01 (*t* test, two-tailed)

We investigated whether ethanol-sensitive miRNAs in the hippocampus were also differentially expressed in serum. Three miRNAs, miR-135a, miR-135b and miR-467b-5p, were significantly up-regulated (≥twofold) in the ethanol-exposed group compared to controls (Fig. 5). Linear regression analysis showed significant linear relationships between the hippocampus and serum for miR-135a (*R*^2^ = 0.84, *P* < 0.01), miR-135b (*R*^2^ = 0.91, *P* < 0.001) and miR-467b-5p (*R*^2^ = 0.60, *P* < 0.05) in ethanol-exposed mice only. The results reveal that, for these miRNAs, serum expression levels could be used as a proxy for hippocampal expression levels in ethanol-exposed offspring.

## Discussion

Alcohol consumption by young women has dramatically increased in recent years raising concern about prenatal alcohol exposure, particularly before pregnancy is confirmed [36]. We have used voluntary maternal consumption of 10 % (v/v) ethanol from fertilisation (0.5 dpc) to 8.5 dpc in C57BL/6J mice to model ethanol exposure during the first 3–4 weeks of human gestation. The estimated peak maternal blood alcohol concentration in this model is 120 mg/dl or 0.12 % [26], approximately twice the legal driving limit in most countries. We previously found that ethanol-exposed offspring had craniofacial dysmorphology at P28–30 [26] and alterations in behaviour [27] in adulthood. Here, we have identified increased *Slc17a6* mRNA and decreased VGLUT2 protein levels in the hippocampi of adult male ethanol-exposed offspring. Transcriptional activation of *Slc17a6* was stochastic and correlated with both reduced promoter DNA methylation and significant enrichment for H3K4me3, suggesting a more open and accessible chromatin conformation. At this stage, we do not know if the observed epigenetic changes are a cause or consequence of the transcriptional changes. The decrease in promoter DNA methylation at *Slc17a6* was modest, changing from an average of 4.5 % methylation per clone in controls to an average of 0.7 % methylation per clone in ethanol-exposed high expressors; however, in vitro reporter studies demonstrated that methylation at a single CpG site was sufficient to repress *Slc17a6* promoter activity. The mechanism by which this occurs is unknown, but appears to be not specific to any particular CpG site. To our knowledge, this is the first report to link promoter DNA methylation and post-translational histone modifications with *Slc17a6* transcription, both normally and in response to an adverse environmental exposure in pregnancy.

Protein levels are generally assumed to correspond with mRNA levels but it is becoming increasingly apparent that mRNA and protein abundances are often only weakly correlated within a tissue [37, 38]. Moreover, the mechanisms underlying these discrepancies are poorly understood. We found that hippocampal VGLUT2 protein levels were consistently decreased in ethanol-exposed offspring regardless of *Slc17a6* mRNA levels. One possible explanation for this is post-transcriptional regulation by miRNAs. Expression profiling in the adult male hippocampus identified 15 candidate ethanol-sensitive miRNAs, ten of which were members of the same family and/or clustered to similar genomic locations, suggesting that the effect of ethanol exposure on miRNA expression is not random. Seven miRNAs representative of each family or genomic location were selected for validation experiments and four miRNAs were confirmed to be significantly up-regulated in ethanol-exposed offspring. Of these, miR-467b-5p was predicted to target *Slc17a6*, among other genes. MicroRNA-467b-5p is positioned in a mouse-specific cluster of up to 65 miRNAs in intron 10 of the *Scm*-*like with four mbt domains 2* (*Sfmbt2*) gene [39]. Five other miRNAs from this cluster were also on our list of fifteen candidate ethanol-sensitive miRNAs. These findings are consistent with Laufer and colleagues, who identified differential expression at three small non-coding RNA clusters, including *Sfmbt2*, in mouse whole brain following prenatal ethanol exposure [23]. In vitro reporter experiments confirmed a specific interaction between the 3′UTR of *Slc17a6* and miR-467b-5p, implicating this miRNA in the down-regulation of hippocampal VGLUT2 in ethanol-exposed mice. Together, the results suggest complex, independent transcriptional and post-transcriptional effects at VGLUT2 in the mouse hippocampus in response to prenatal ethanol exposure. Although the impact of miR-467b-5p would not be reproduced in humans, our findings show that such a mechanism is possible in the hippocampus and could involve other ethanol-sensitive miRNAs.

Of the remaining validated ethanol-sensitive miRNAs, miR-135a and miR-135b are well-conserved and predicted to target *Complexin 1* and *Complexin 2* (TargetScan, Release 6.2), which are involved in modulating neurotransmitter release [40]. *Complexin 1* and *Complexin 2* mRNAs were not differentially expressed between groups in our initial microarray-based screen of gene expression; however, it is possible that these miRNAs affect translation and protein levels are changed. MicroRNA regulation of target protein translation (with no effect on mRNA levels) has previously been reported in the placenta in a mouse model of intrauterine calorie restriction [41]. Analysis of putative target genes for miR-487b did not reveal any candidates that are known to play a role in hippocampal function.

There is increasing interest in the use of circulating miRNAs in the diagnosis of human disease, particularly cancer [42–44]. Recent investigation of an ovine model of binge ethanol exposure throughout pregnancy identified a number of plasma miRNAs in newborn lambs that were sensitive and specific indicators of prenatal ethanol exposure, and were predicted to target pathways involved in growth [45]. We found that the three ethanol-sensitive miRNAs with the highest average expression fold changes in the hippocampus, including the well-conserved miR-135a and miR-135b, were also significantly up-regulated in serum. Interestingly, expression levels in both tissues were significantly linearly correlated in the same animal suggesting that, for these miRNAs, serum expression could be used as a surrogate for expression levels in the hippocampus.

## Conclusions

Prenatal ethanol exposure affects hippocampal gene expression and epigenetic state at multiple levels. Further, these effects are observed following a relatively moderate exposure early in pregnancy, and only in adult male offspring. Our findings are consistent with the idea that some of the cognitive and behavioural phenotypes observed in foetal alcohol spectrum disorders may be due to altered epigenetic and/or miRNA-mediated control of glutamate neurotransmission in the hippocampus. Although further work is needed in both mice and humans, our findings also suggest that circulating miRNAs could be used as noninvasive biomarkers of in utero ethanol exposure as well as hippocampal dysfunction.

## Methods

### Animals and prenatal ethanol exposure

Animal work was conducted in accordance with the Australian code for the care and use of animals for scientific purposes, and was approved by an Animal Ethics Committee at The University of Queensland (MMRI/120/12/NHMRC). Prenatal ethanol exposure in inbred C57BL/6J mice was performed as described previously [26]. The model is based on voluntary maternal consumption of 10 % (v/v) ethanol from fertilisation to 8.5 dpc. Briefly, adult (8-week-old) C57BL/6J males and C57BL/6J females (6–7 weeks) were purchased from the Animal Resources Centre (Perth, Australia) and habituated to a 12-h light/12-h dark cycle for a week before mating. Males were paired with a single, nulliparous female overnight and females were checked each morning for the presence of a vaginal plug (defined as 0.5 dpc). At 0.5 dpc, the male and female were separated and females were randomly assigned to either the ethanol-exposed group [10 % (v/v) ethanol] or control group (water). Pregnant females were allowed free access to both food and liquid, and the volume of liquid consumed was measured to the nearest 0.2 ml every 24 h. Following 8 days of exposure, ethanol-exposed females were given water for the remainder of the experiment. Female body weight was measured at 0.5 and 8.5 dpc.

### Microarray-based analysis of gene expression

Hippocampi were dissected from male ethanol-exposed and control offspring (*n* = 6 per group) at P87, snap-frozen in liquid nitrogen and stored at −80 °C. Total RNA was extracted using the RNeasy Plus Mini kit (Qiagen, Netherlands). For each sample, 500 ng of total RNA was used for complementary RNA (cRNA) synthesis using the TotalPrep RNA Amplification kit (Illumina, USA) and 1.5 µg of cRNA was then hybridised to MouseWG-6 v2.0 Expression BeadChips (Illumina, USA) at 58 °C for 16 h according to the manufacturer’s instructions. Data were quantile normalised using Genome Studio v1.9.0 (Illumina, USA) and differential expression was determined using GeneSpring GX11 (Agilent, USA) on default settings. Genes with uncorrected *P* values less than 0.05 and expression fold changes greater than 1.5 were selected for validation experiments.

### Availability of supporting data

The microarray data sets supporting the results of this article are available in the Gene Expression Omnibus (GEO) repository under accession number GSE60000 (http://www.ncbi.nlm.nih.gov/geo/).

### qPCR validation of differential gene expression

RNA (250 ng) was reverse transcribed in a 20 µl reaction using 100 ng of random hexamer primers (Life Technologies, USA), 500 µM dNTP mix, 1× first strand buffer, 5 mM DTT, 40 units of RNaseOUT (Life Technologies, USA) and 200 units of SuperScript III Reverse Transcriptase (Life Technologies, USA). The cDNA was then diluted 1 in 10 and 4 µl was used in the qPCR with 1× SYBR green MasterMix (Life Technologies, USA), 0.3 µM of forward/reverse primers (Additional file 1: Table S3) and 0.2 µl of ROX dye (Life Technologies, USA). Cycling parameters were 50 °C for 5 min, followed by 40 cycles of denaturation at 95 °C for 15 s and annealing and extension at 60 °C for 1 min. Each reaction was performed in triplicate. *Gapdh* and *Hprt1* were used as housekeepers, and similar results were obtained with both genes. Analysis was performed using the 2^−ΔΔCT^ method.

### Clonal bisulphite sequencing

A total of 1 µg of genomic DNA was converted with sodium bisulphite using the EpiTect Bisulfite kit (Qiagen, Netherlands) according to the manufacturer’s instructions. A region (BS1) extending from −144 to −20 bp relative to the transcriptional start site identified by Li and colleagues in the mouse brain [31] was then amplified using a semi-nested PCR strategy. The first round PCR was conducted in a 50 µl reaction that contained 3 µl of converted genomic DNA, 1× BIOTAQ NH_4_ buffer, 1.5 mM MgCl_2_, 200 µM dNTPs, 0.3 µM of each primer (BS_F and BS_R1, Additional file 1: Table S3) and 1 U BIOTAQ DNA Polymerase (Bioline, UK). Cycling parameters involved denaturation at 94 °C for 5 min followed by 29 cycles of denaturation at 94 °C for 1 min, annealing at 58 °C for 1 min, and extension at 72 °C for 1 min. A final extension step was carried out at 72 °C for 3 min. First round PCR product (1 µl) was used as template for a second round PCR using primers BS_F and BS_R2 (Additional file 1: Table S3) and the same reaction and cycling conditions as the first round. Two independent semi-nested PCRs were performed per sample to reduce the impact of PCR bias on the results [46]. PCR products were gel-purified, subcloned into the pGEM-T vector (Promega, USA) and transformed into *E. coli* DH5α competent cells according to standard protocols. Two to four clones were sequenced per PCR and were analysed using BiQ Analyzer with default settings [47].

### In vitro reporter assays of promoter function

The *Slc17a6* promoter (−144 to +68 bp) was amplified from mouse hippocampal genomic DNA using the primers PM_F and PM_R (Additional file 1: Table S3) and ligated to the pGL4.14 [*luc2*/Hygro] vector (Promega, USA) using *Sfi*I restriction enzyme sites. A CpG methyltransferase (*M.SssI*), *Hpa*II methyltransferase or *Hha*I methyltransferase (New England Biolabs, USA) were used either alone or in combination to methylate the promoter-containing construct and vector only according to the manufacturer’s instructions. pGL4.14-based plasmids (45 ng) and pGL4.74[*hRluc*/TK] (5 ng) were transiently transfected into CAD cells using Lipofectamine 2000 (Life Technologies, USA) according to the manufacturer’s protocol. CAD cells were grown in DMEM:HAMS F12 (1:1) with 8 % foetal bovine serum and 2 mM glutamine. pGL4.74[*hRluc*/TK] contains *Renilla* luciferase and was used to control for transfection efficiency. Transfections were performed in quadruplicate. Luciferase assays were carried out 36 h after transfection using the Dual-Glo luciferase assay system (Promega, USA) according to the manufacturer’s instructions.

### ChIP-qPCR

ChIP-qPCR was performed using the ChIP-IT High-Sensitivity Kit and ChIP-IT qPCR Analysis Kit (Active Motif, USA) according to the manufacturer’s instructions. Chromatin was prepared from the pooled hippocampi of twelve males per group. *Slc17a6* ChIP-qPCR primers, ChIP_F and ChIP_R, are listed in Table S3 (Additional file 1). The H3K4me3 and H3K27me3 antibodies, Mouse Negative Control Primer Set 1, Positive Control Primer Set *Gapdh*-*2* and Positive Control Primer Set *Pax*-2 were all purchased from Active Motif. Reactions were performed in triplicate. Data were analysed using an Excel-based ChIP-IT qPCR Analysis Spreadsheet according to the manufacturer’s instructions [48].

### Western blotting

Individual hippocampi were homogenised in a urea protein lysis buffer, centrifuged at 13,000 rpm for 5 min at 4 °C and the supernatants were and stored at −80 °C. Total protein concentration was determined using the BCA Protein Assay (Thermo Fisher Scientific, USA) following the manufacturer’s instructions. 10 µg of total protein was electrophoresed on a NuPage 4–12 % Bis–Tris gel (Life Technologies, USA) and transferred onto PDVF membrane using the Hoefer miniVE (Hoefer, USA). The membrane was incubated in Odyssey blocking buffer (LI-COR Biosciences, USA) at room temperature for 90 min, followed by incubation with a primary guinea pig polyclonal anti-VGLUT2 antibody (135404; Synaptic Systems, Germany) diluted with 5 ml Odyssey blocking buffer at a ratio of 1:1000 and 0.1 % Tween 20 (Sigma-Aldrich, USA) at 4 °C overnight with gentle rolling. The membrane was then washed four times with PBS containing 0.1 % Tween 20 (5 min each at room temperature). A fluorescent-labelled secondary antibody, IRDye 680RD Donkey anti-guinea pig (LI-COR Biosciences, USA) diluted with Odyssey blocking buffer at a ratio of 1:10,000, supplemented with 0.01 % SDS, was then incubated with the membrane in the dark at room temperature for 45 min. The membrane was washed again and scanned using the Odyssey system (LI-COR Biosciences, USA). After scanning, the membrane was rinsed with blocking buffer and incubated with a primary mouse monoclonal anti-GAPDH antibody (MAB374; Millipore, USA) at room temperature for an hour. A different fluorescent-labelled secondary antibody, IRDye 800RD Donkey anti-mouse (LI-COR Biosciences, USA), was incubated with the membrane at room temperature for 45 min followed by four washes as mentioned above. The membrane was re-scanned and VGLUT2 levels were calculated relative to GAPDH levels.

### MicroRNA profiling

Small RNA was isolated from the hippocampus using the miRNeasy mini kit (Qiagen, Netherlands). RNA (200 ng) was reverse transcribed using the miScript II RT kit (Qiagen, Netherlands) and genome-wide miRNA profiling was conducted using the miScript miRNA PCR Array (MIMM-3216ZE-12; Qiagen, Netherlands) according to the manufacturer’s instructions. A total of 944 different mature miRNAs as well as six housekeeping genes were assayed. Data were normalised to the average Ct of the two most stably expressed housekeepers: *RNU6*-*2* and *SNORD95*, and analysed by the 2^−ΔΔCT^ method using an Excel-based miScript PCR Array Data Analysis Template [49].

### TaqMan qPCR validation of differential miRNA expression

cDNA was prepared using reverse transcription primers provided with TaqMan MicroRNA Assays (Life Technologies, USA) and the TaqMan MicroRNA Reverse Transcription kit (Life Technologies, USA) according to the manufacturer’s instructions. The cDNA was then diluted 10 times with RNase-free water and 4.5 μl was mixed with 5 μl TaqMan Universal Master Mix II, no UNG (Life Technologies, USA) and 1× TaqMan MicroRNA Assay to obtain a 10 µl reaction. PCR was performed on a ViiA 7 (Applied Biosystems, USA) using 10 min denaturation at 95 °C, followed by 40 repeats of denaturation at 95 °C for 15 s and annealing and extension at 60 °C for 60 s. Reactions were performed in triplicate. Data were normalised to *RNU6*-*2* and relative expression was calculated by the 2^−ΔΔCT^ method.

### Luciferase reporter assay of miRNA–target mRNA interaction

Plasmid constructs containing the putative miR-467b-5p target site (position 374–380 of *Slc17a6* 3′UTR, NCBI:NM_080853) (Target) or a mutated target site (Scramble), containing seven mismatches in the seed region, were engineered using the *pmir*GLO vector system (Promega, USA) according to the manufacturer’s instructions. *pmir*GLO contains both a firefly luciferase reporter gene (*luc2*) and a *Renilla* luciferase reporter gene (*hRluc*-*neo fusion*). Inserts with *Pme*I (5′) and *Xba*I (3′) sticky ends were generated by annealing 2 μg of sense oligonucleotides and 2 μg of antisense oligonucleotides (Additional file 1: Table S3) in 46 μl of annealing buffer at 37 °C for 15 min after denaturation at 90 °C for 3 min. Annealed products, Target or Scramble, were ligated to linearized pmirGLO that had been digested with *Pme*I and *Xba*I. The constructs or vector only (50 ng) were transfected into 2 × 10^4^ CAD cells using Lipofectamine 2000 (Life Technologies, USA) according to the manufacturer’s protocol. Co-transfection with a miR-467b-5p mimic or an inhibitor (3.3 pmol) was performed using the same conditions. Transfected cells were cultured for 24 h and then subjected to a luciferase assay using the Dual-Glo luciferase assay system (Promega, USA) according to the manufacturer’s instructions. Assays were done in quadruplicate. Firefly luciferase activity was normalised to *Renilla* luciferase activity.

### Analysis of alcohol-sensitive miRNAs in serum

Serum was isolated from 500 μl of whole blood by centrifugation at 8000 rcf for 15 min. Serum samples (100 μl) were mixed with 3.5 µl of a synthetic spike-in control, *Caenorhabditis elegans* microRNA 39 (Syn-cel-miR-39) (1.6 × 10^8^ copies/µl), before isolation of total RNA using miRNeasy mini kit according to the serum RNA extraction protocol (Qiagen, Netherlands). Total RNA (170 ng) was reverse transcribed into cDNA using the TaqMan microRNA reverse transcription kit (Life Technologies, USA) following manufacturer’s instructions. Pre-amplification was performed using 1× TaqMan PreAmp Master Mix (Life Technologies, USA), 0.0075× pre-amplification primer pool and 2.5 μl of RT products. Cycling parameters were 95 °C for 10 min, 55 °C for 2 min and 72 °C for 2 min, followed by 12 cycles of denaturation at 95 °C for 15 s and annealing and extension at 60 °C for 4 min with a final deactivation at 99.9 °C for 10 min. To amplify the miRNAs of interest, 2.5 µl of pre-amplification products (diluted 40 times) was amplified using 1× TaqMan microRNA assay in the presence of 1× TaqMan Universal Master mix II (No AmpErase UNG) (Life Technologies, USA) following manufacturer’s instructions. Reactions were performed in triplicate. Data were normalised using Syn-cel-miR-39 and analysed using the 2^−ΔΔCT^ method.

### Statistics

All statistical analyses were conducted using R [50].

## Additional file

10.1186/s13072-015-0032-6 **Figures S1**–**S6** and **Tables S1**–**S3**. **Figure S1.** Maternal liquid consumption (A) and % weight gain (B) over the 8-day exposure period. (C) Litter size at P21. **Figure S2.** *Slc17a6* expression in the female hippocampus at P87. **Figure S3.** *Slc17a6* expression in the male hippocampus at P21 and P120. **Figure S4.** *Slc17a6* promoter DNA methylation in the hippocampus at P21. **Figure S5.** The impact of *Slc17a6* promoter methylation on luciferase activity in vitro. **Figure S6.** MicroRNA-467b-5p targets the 3′UTR of *Slc17a6* in an in vitro reporter assay. **Table S1.** Differentially expressed genes in adult male hippocampi. **Table S2**. Candidate ethanol-sensitive miRNAs in the hippocampus. **Table S3.** Oligonucleotides used in this study.

## Abbreviations

3′UTR: 3′ untranslated region
5′UTR: 5′ untranslated region
CAD: Cath.a-differentiated
ChIP: chromatin immunoprecipitation
cRNA: complementary RNA
Ctrl: control
dpc: days post coitum
EtOH: ethanol
H3K4me3: histone H3 lysine 4 trimethylation
H3K27me3: histone H3 lysine 27 trimethylation
miRNA: microRNA
mRNA: messenger RNA
MTs: methyltransferases
P: postnatal day
qPCR: quantitative PCR
RT: reverse transcription
*Slc17a6*: solute carrier family 17 member 6
TSS: transcriptional start site
VGLUT2: vesicular glutamate transporter 2
