# Supplementary material for: Prenatal ethanol exposure alters adult hippocampal VGLUT2 expression with concomitant changes in promoter DNA methylation, H3K4 trimethylation and miR-467b-5p levels
Source: Epigenetics Chromatin. 2015 Sep 28;8:40. doi: 10.1186/s13072-015-0032-6 (PMC4587775; doi:10.1186/s13072-015-0032-6)
Supplement: Supplementary file 1 — 10.1186/s13072-015-0032-6 Figures S1–S6 and Tables S1–S3. Figure S1. Maternal liquid consumption (A) and % weight gain (B) over the 8-day exposure period. (C) Litter size at P21. Figure S2. Slc17a6 expression in the female hippocampus at P87. Figure S3. Slc17a6 expression in the male hippocampus at P21 and P120. Figure S4. Slc17a6 promoter DNA methylation in the hippocampus at P21. Figure S5. The impact of Slc17a6 promoter methylation on luciferase activity in vitro. Figure S6. MicroRNA-467b-5p targets the 3′UTR of Slc17a6 in an in vitro reporter assay. Table S1. Differentially expressed genes in adult male hippocampi. Table S2. Candidate ethanol-sensitive miRNAs in the hippocampus. Table S3. Oligonucleotides used in this study. [file 13072_2015_32_MOESM1_ESM.pdf]

Supplementary Information

Figure legends

Figure S1. Maternal liquid consumption per day *(A)* and % weight gain *(B)* over the 8-day exposure period (0.5-8.5 dpc). *(C)* Litter size at P21. EtOH: ethanol-exposed mice. Data is shown as mean and standard deviation.

Figure S2. *Slc17a6* expression in the female hippocampus at P87. EtOH: ethanol-exposed mice. Data were normalised to *Hprt1* and is plotted as mean and standard deviation.

Figure S3. *Slc17a6* expression in the male hippocampus at P21 and P120. EtOH: ethanol-exposed mice. Data were normalised to *Hprt1* and is plotted as mean and standard deviation. **P<0.01 (t-test, two-tailed).

Figure S4. *Slc17a6* promoter DNA methylation (BS1, -144 to -20 bp) in the hippocampus at P21. Filled and open circles represent methylated and unmethylated CpGs, respectively. Each line of nine CpGs represents the DNA methylation state of one allele in one cell. 38 clones from controls (18 mice) and 47 clones from ethanol-exposed mice (EtOH, 20 mice) are shown. A graph of % DNA methylation per clone is also shown. Data is presented as mean and standard deviation. At P21 *Slc17a6* expression was equivalent in ethanol-exposed and control mice.

Figure S5. The impact of *Slc17a6* promoter methylation (CpGs 1-10, -144 to +68 bp) on luciferase activity *in vitro*. The results of three independent experiments in Neuro 2a cells are shown. Data are presented as mean and standard deviation. **P<0.01 (t-test, two-tailed).

Figure S6. MicroRNA-467b-5p targets the 3’UTR of *Slc17a6* in an *in vitro* reporter assay. Luminescence is shown for vector only, vector containing the 3’UTR of *Slc17a6* (Target) and vector with a mutated *Slc17a6* 3’UTR (Scramble) under three different co-transfection conditions: no co-transfection (None), addition of a miR-467b-5p mimic (Mimic) or addition of a miR-467b-5p inhibitor (Inhibitor). Assays were done in quadruplicate, and the results

from three independent experiments in Neuro 2a cells are shown. Data are shown as mean and standard deviation. *P<0.05 (*t*-test, two-tailed).

Figure S1.

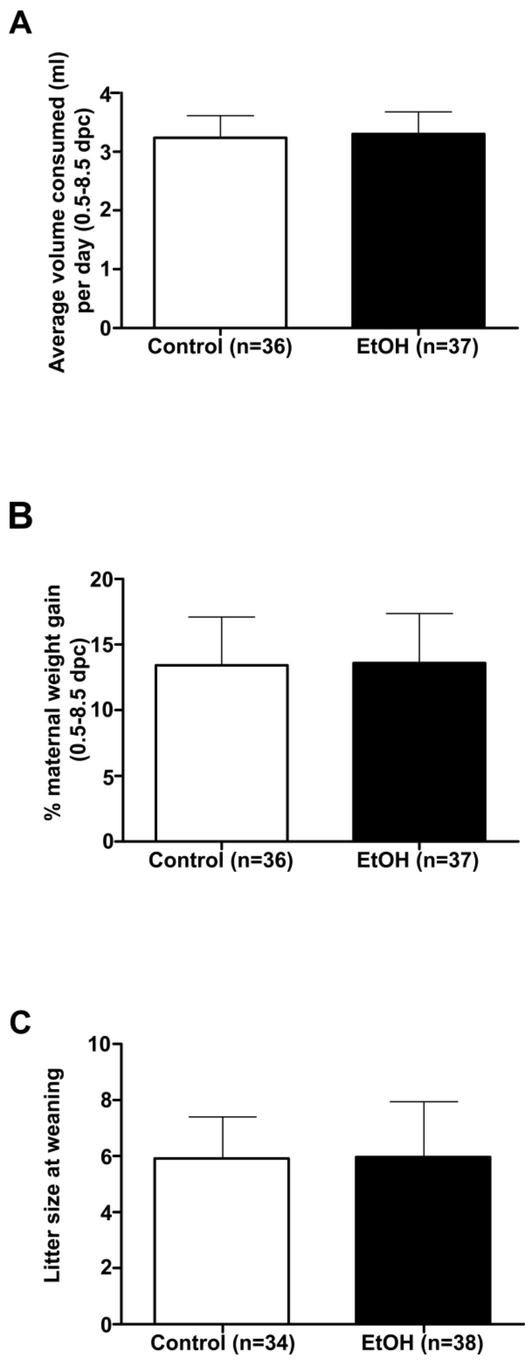
A
Average volume consumed (ml) per day (0.5-8.5 dpc)
0
1
2
3
4
Control (n=36)
EtOH (n=37)
B
% maternal weight gain (0.5-8.5 dpc)
0
5
10
15
20
Control (n=36)
EtOH (n=37)
C
Litter size at weaning
0
2
4
6
8
10
Control (n=34)
EtOH (n=38)

Figure S2.

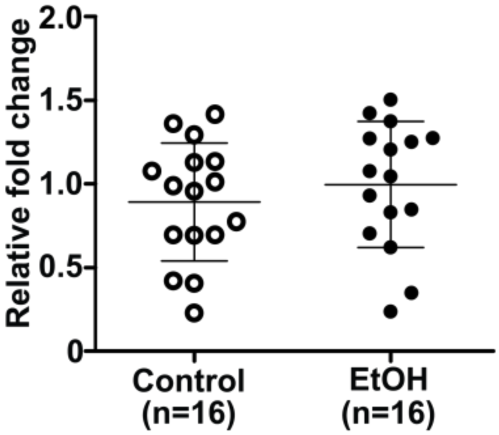

Figure S3.

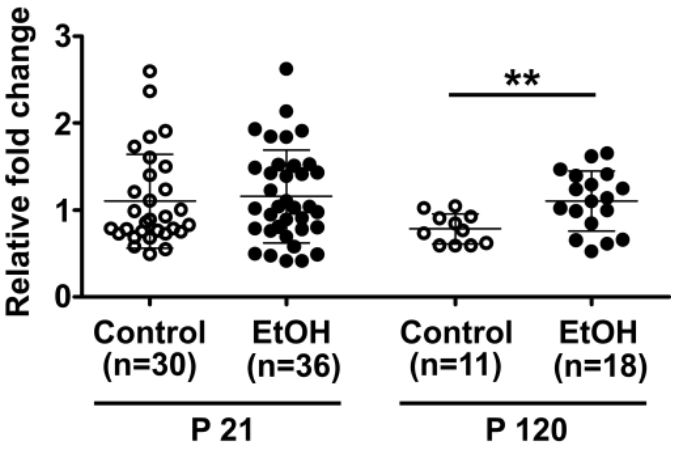
Relative fold change
3
2
1
0
**
Control
(n=30)
EtOH
(n=36)
Control
(n=11)
EtOH
(n=18)
P 21
P 120

Figure S4.

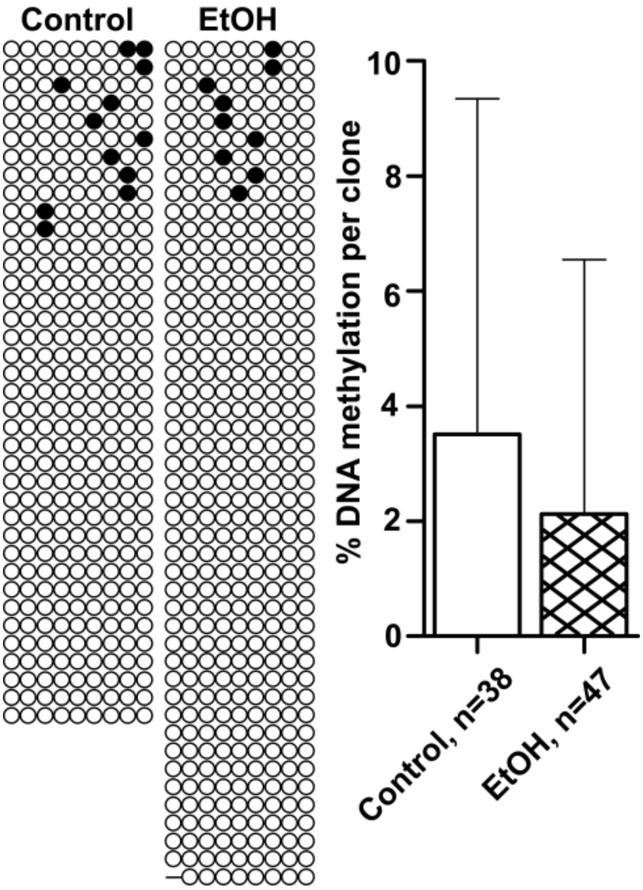
Control
EtOH
10
8
6
4
2
0
% DNA methylation per clone
Control, n=38
EtOH, n=47

Figure S5.

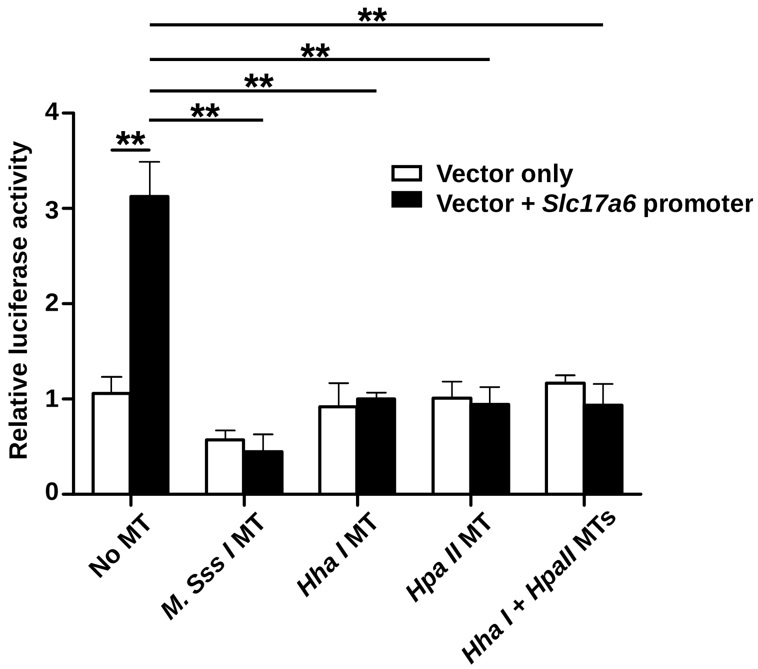
**
**
**
**
**
4
3
2
1
0
Relative luciferase activity
Vector only
Vector + Slc17a6 promoter
No MT
M. Sss I MT
Hha I MT
Hpa II MT
Hha I + HpaII MTs

Figure S6.

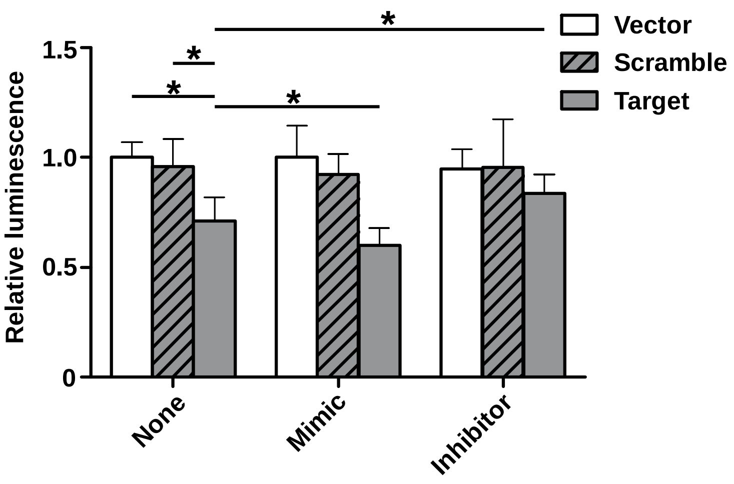
Relative luminescence
1.5
1.0
0.5
0
*
*
*
*
Vector
Scramble
Target
None
Mimic
Inhibitor

Table S1. Differentially expressed genes in adult male hippocampi

Gene[†]	*P*-value[‡]	Fold Change[§]	Illumina Probe ID
Indolethylamine N-methyltransferase (Inmt)	0.03	-1.59	ILMN_1231445
Melanoma inhibitory activity 1 (Mia1)	0.05	-1.55	ILMN_2777087
Solute carrier family 17 member 6 (Slc17a6)	0.02	1.56	ILMN_2773047
	0.02	1.55	ILMN_2922321
Teashirt zinc finger family member 2 (Tshz2)	0.03	1.51	ILMN_1256408

[†]Gene information was obtained from UCSC mouse genome database (Feb. 2006 (NCBI36/mm8)); [‡]Indicates uncorrected *P*-Value; [§]Positive and negative values indicate up-regulation and down-regulation, respectively.

Table S2. Candidate ethanol-sensitive microRNAs in the hippocampus

ID	*P*-value	FC*	Family	Reason for exclusion from qPCR validation experiments
mmu-miR-669a-5p	1.99E-05	1.539	miR-467	Clustered with miR-467b
mmu-miR-467a*	2.87E-05	1.866	miR-467	Clustered with miR-467b
mmu-miR-3097-3p	8.78E-05	2.339	miR-3097	TaqMan assay not available
mmu-miR-135a	9.94E-05	1.745	miR-135	-
mmu-miR-467b-5p	0.00011	2.338	miR-467	-
mmu-miR-669b*	0.000114	2.357	miR-467	Clustered with miR-467b
mmu-miR-135b	0.00014	1.877	miR-135	-
mmu-miR-487b	0.000191	1.977	miR-154	-
mmu-miR-450b-5p	0.000257	2.784	miR-450	-
mmu-miR-466c-5p	0.00026	2.128	miR-466	Clustered with miR-467b
mmu-miR-592	0.000333	1.815	miR-592	TaqMan assay not available
mmu-miR-467e	0.000394	1.771	miR-467	Clustered with miR-467b
mmu-miR-380-5p	0.000412	1.993	miR-379	-
mmu-miR-369-3p	0.000427	2.007	miR-154	same family as miR-487b
mmu-miR-335-3p	0.000482	1.737	miR-335	-

FC: expression fold-change. *Positive values indicate up-regulation of microRNAs.

Table S3. Oligonucleotides used in this study

ID	Sequence (5'-3')	Experiment
Slc17a6_F	gcggaggcaaagttatcaag	qPCR
Slc17a6_R	cctggaatctgggtgatgat	qPCR
Gapdh_F	tcgttgatggcaacaatctc	qPCR
Gapdh_R	cgtcccgtagacaaaatggt	qPCR
BS_F	gttaggttttatggaaggttttttt	Bisulphite PCR, Forward
BS_R1	atcaaaactcataactctcaaaacc	Bisulphite PCR, Reverse 1
BS_R2	aacttcctaccttaacataatctcctc	Bisulphite PCR, Reverse 2
PM_F	ggcctaactggcccggcgctgcctaaaccaca	Amplify the Slc17a6 promoter
PM_R	ggccgccgaggccgagctcatagctctcagaa	Amplify the Slc17a6 promoter
ChIP_F	ccaaggttccttagcttcct	ChIP qPCR
ChIP_R	ttgcgaacgtgagtgaataa	ChIP qPCR
Slc_TF	aaactagcggccgctagt**tggatcatgcaaactgcactta**t	+ strand, target
Slc_TR	ctaga**taagtgcagtttgcatgatcca**actagcggccgctagttt	- strand, target
Slc_MF	aaactagcggccgctagt**t*taa*t*acgat*gagct*atgaggc***t	+ strand, scramble
Slc_MR	ctaga***gcctcat*agct*catcgt*at*taa*a**actagcggccgctagttt	- strand, scramble

Underlining indicates restriction enzyme recognition sites: *Sfi I* for PM_F and PM_R; *Not I* for Slc_TF, Slc_TR, Slc_MF and Slc_MR. Bold characters indicate the putative target site of miR-467b-5p and bold italic letters are the arbitrary mismatches.
